# Retraction: Perennial plants and traditional medicine: Assessing the socio-demographic impacts on traditional remedies

**DOI:** 10.1371/journal.pone.0346206

**Published:** 2026-04-01

**Authors:** The PLOS One Editors

After the publication of this article [1], several concerns were raised about the reliability and integrity of information reported in the article and provided to the journal at submission. Additionally, the corresponding author YZ contacted the journal editors to request retraction of the article, stating that an internal review had identified errors in the data analysis and concerns about ethical compliance.

The *PLOS One* Editors followed up with the authors and also consulted The Islamia University of Bahawalpur, and note the following issues:

- Documentation of approval for the study from The Islamia University of Bahawalpur Institutional Human Ethics Committee was provided to the editors at the time of initial submission of the manuscript. However, following review of institutional records, The Islamia University of Bahawalpur stated that the approval letter was not issued by the Institutional Human Ethics Committee of the Islamia University of Bahawalpur.
- The funding grants reported in the Funding statement of the article appear to be inconsistent with the study’s topic. Upon follow-up, the corresponding author HQ stated that the listed funding supported general institutional research capacity and did not influence the study’s design or results.
- Issues in the reported data, including inconsistencies in the demographic percentages and totals in Table 1. Upon follow-up, the corresponding author HQ stated that inconsistencies in Table 1 arise from rounding of percentages and indicated the values reported in tables accurately reflect the patterns of informant responses.

In light of these concerns, which call into question the integrity and reliability of the published results, the *PLOS One* Editors retract this article.

YZ and HQ did not respond to the final editorial decision. XW, XC, TA, and A either did not respond directly or could not be reached.
